# Examining the double-edged effects of digital literacy on the social integration of retirement migrants

**DOI:** 10.3389/fpubh.2025.1470319

**Published:** 2025-03-31

**Authors:** Kaishuai Wang, Yiming Feng, Xue Qin, Yumai Hu

**Affiliations:** ^1^Department of Tourism and Hospitality Management, School of Management, Zhejiang University, Hangzhou, Zhejiang, China; ^2^School of Art and Archaeology, Zhejiang University, Hangzhou, Zhejiang, China; ^3^School of Management, Guizhou University, Guiyang, Guizhou, China; ^4^School of Economy and Management, Guizhou Qiannan College of Science and Technology, Huishui, Guizhou, China; ^5^Faculty of Science and Engineering, University of Nottingham Ningbo China, Ningbo, China

**Keywords:** retirement migrants, social integration, digital literacy, tourist empowerment, smartphone addiction

## Abstract

**Introduction:**

The retiree group tend to suffer “digital gap” and “digital indulgence” at the same time. This study aims to examine how such double-edged effects will shape the social integration of retirement migrants. Based on the Self-Determination Theory and the Uses and Gratifications Theory, a conceptual framework was proposed where the impact of digital literacy is mediated by tourist empowerment and smartphone addiction.

**Methods:**

This framework was tested using survey data gathered from 369 Chinese retirement migrants. Data were collected using convenience sampling and analyzed using SPSS 23.0 and AMOS 23.0.

**Result:**

Findings reveal the following key insights: (1) Digital literacy significantly enhances the social integration of retirement migrants; (2) Tourist empowerment acts as a mediator in the positive correlation between digital literacy and social integration; (3) Smartphone addiction detrimentally influences the lifestyle habits dimension of social integration among retirement migrants.

**Discussion:**

This study was amongst the first to approach the double-edged effects of digital literacy, and the findings could be conducive to improving the welfare of retirees.

## 1 Introduction

Digitalization exerts a pervasive influence across diverse spheres of individuals’ professional and personal lives. Activities like travel, shopping, healthcare, and administrative duties are increasingly reliant on digital technologies (1). Older adults emerge as a vulnerable cohort within the nexus of digitalization and aging. Hence, augmenting their social integration in the digital epoch stands as a matter of paramount significance. In the tourism sector, the intersection of aging and digitalization is evidenced through the influence of digital literacy on the social integration of retirement migrants. The demographic shift toward an aging population has resulted in a notable rise in the population of retirement migrants (2, 3). Key determinants affecting social integration encompass institutional, community, familial, and individual dimensions (4). Within the contemporary digital landscape, the role of individual-level digital literacy in shaping social integration is progressively gaining prominence (5). Consequently, there exists a compelling need for comprehensive exploration into the realms of digital literacy and social integration concerning retirement migrants.

The impact of digital literacy on social integration is a subject of contention, with two contrasting viewpoints necessitating further scrutiny. Regarding the influence of digital literacy, two fundamentally divergent perspectives emerge “optimism” and “pessimism” (1). Proponents of the optimistic view contend that “technology empowerment” enables marginalized groups to engage in digital social interactions, compensates for familial voids, and enhances mental wellbeing (1). For instance, studies suggest that the utilization of smartphones can enhance the quality of life and overall wellbeing of older adults (6). Conversely, advocates of the pessimistic stance argue that the “digital divide” stemming from varying levels of digital literacy is insurmountable, exacerbating the inherent disadvantages faced by digitally marginalized groups in the digital realm, thereby intensifying their sense of relative deprivation (1). While smartphones offer convenience and user-friendliness, they also present a potential “attraction trap, “potentially leading to smartphone addiction (7). Consequently, the use of smartphones has been observed to have a dual impact on psychosocial factors (8). Hence, the inquiry into whether digital literacy exerts a dual influence on the social integration of retirement migrants warrants further investigation.

This study aims to model and test the double-edged effects of digital literacy on the social integration of retirement migrants. A conceptual framework was proposed where the impact of digital literacy is mediated by tourist empowerment and smartphone addiction. This framework was tested using survey data gathered from 369 Chinese retirement migrants. This study makes three main contributions: (1) it introduces digital literacy as a new explanatory variable for understanding social integration; (2) it reveals the double-edged effects of digital literacy on social integration; and (3) it provides important practical implications for enhancing older adults social integration. These contributions advance our understanding of retirement migrants’ social integration in the digital.

The remainder of this paper is structured as follows: first, the literature review section defines the research subject and systematically reviews previous studies on key variables including digital literacy, tourist empowerment, smartphone dependency, and social integration. Building on this literature, the theoretical framework and hypotheses section develops a conceptual model based on Self-Determination Theory and Uses and Gratifications Theory, and proposes research hypotheses. Subsequently, the methodology section details the data sources, measurement scales, data collection procedures, and analytical tools employed. Following this, the results section presents empirical tests of the hypothesized model. Next, the discussion section engages our findings with existing literature. Finally, the conclusion section summarizes the main findings, discusses theoretical contributions and practical implications, and addresses limitations and future research directions.

## 2 Literature review

### 2.1 Retirement migrants

Retirement migrants are individuals who permanently or temporarily relocate in pursuit of a “good life” (9, 10). Typically, these migrants are over the age of 50 or 60, depending on the retirement standards of each country, and have exited the labor market or begun receiving pensions (2, 11). They occupy a space between tourists and permanent residents, aiming for a happy life while placing significant emphasis on the tourism experience (2). Their duration of stay at the destination is flexible, reflecting diverse mobility patterns that range from a few months each year to several years (9). As a distinctive form of tourism mobility within a unique demographic, retirement migrants have garnered significant attention from tourism scholars (12, 13).

The phenomenon of retirement migrants first emerged in North America during the 1950s (14). Since then, retirement migration has evolved into a global spatiotemporal phenomenon (15, 16). Unlike retirees from Europe and North America who often choose to retire abroad (9, 16), Chinese retirement migrants primarily relocate to domestic destination cities such as Zhuhai (2) and Sanya (3, 126). With the acceleration of population aging in China, the number of retirement migrants is rapidly increasing, leading to various social issues in tourist destinations. Enhancing the social integration of retirement migrants can effectively mitigate social conflicts (2).

### 2.2 Social integration

Social integration denotes the extent of an individual’s connections within broad social relationships and their active participation in social life (17). Social integration is not a static or unidimensional concept; rather, it is dynamic, progressive, multidimensional, and interactive (18). Scholars have proposed various measurement indicators from both macro and micro levels, encompassing subjective and objective perspectives (19–21). For instance, Yang (18) categorizes social integration into four dimensions: economic integration, cultural acceptance, behavioral adaptation, and identity recognition. The integration process for lifestyle migrants encompasses three primary dimensions: emotional attachment, cultural adaptation, and social participation (22).

The social integration of retirement migrants in new living environments represents a relatively new area of research (23). Studies specifically addressing the social integration of retirement migrants generally emphasize interpersonal interactions, lifestyle habits, cultural acceptance, and psychological distance (2). Over time, as these migrants engage in more extensive and meaningful social interactions, they experience a continuously deepening and dynamically evolving process of social integration (24).

This study defines the social integration of retirement migrants as the process of adapting to local life and mutual adaptation among retirement migrants, including three dimensions: lifestyle habits, interpersonal interactions, and activity participation. The factors influencing the social integration of older migrants can be categorized into four levels: individual factors, family factors, social support factors, and policy assurance factors (25). In the digital age, the advent of digital technologies, exemplified by smartphones and social media, has profoundly transformed modes of communication and interaction (57). Consequently, the relationship between digital literacy and the social integration of older adults warrants further investigation.

### 2.3 Digital literacy

Digital literacy is defined as the capability to access and utilize information via digital devices (26, 27). This concept extends beyond basic technical skills to include the ability to evaluate, utilize, and produce digital content (28). Initially, literacy pertained to familiarity with computers, which evolved into what is known as computer literacy (29–31). Subsequently, with the proliferation of the internet, information and communication technologies, online platforms, and digital media, the term “computer literacy” further transformed into “digital literacy” (29). Associated terms such as ICT literacy, network literacy, information literacy, and electronic literacy have been discussed in the scholarly literature as interchangeable with digital literacy (32). The concept of digital literacy is increasingly prevalent in the 21st century (33). For instance, Reddy et al. (34) introduced the digital literacy framework for the South Pacific region.

In the contemporary era of mobile internet, the use of smartphones and social media has become emblematic of digital engagement (57). Smartphones, characterized by their portability, immediacy, and convenience, have become an indispensable element of contemporary daily life (35, 36). They fulfill a multitude of needs including information access, instant communication, social interaction, and entertainment (37, 38). Social media platforms offer tourists the opportunity to receive and share travel experiences, as well as disseminate information about destinations and amenities (39). Additionally, various hospitality and tourism enterprises utilize social media to enhance their operations and customer engagement (40).

### 2.4 Tourist empowerment

Empowerment refers to the initiatives undertaken by individuals or groups to control their destiny by enhancing their capabilities (i.e., internal power) and influencing their environment (i.e., external response) (41, 42). Empowerment transforms individuals into proactive agents who act in alignment with their values and interests (43). In the digital age, devoid of the constraints of time and place, anyone with internet access can more readily access vast amounts of information and disseminate information to millions at unprecedented speeds (44, 45). Consequently, consumer power has evolved from being based on individual needs and information to being rooted in networks and crowds (46).

Empowerment research within the tourism sector can be broadly categorized into three primary themes: the empowerment of destination residents, the empowerment of tourism practitioners, and the empowerment of tourists (47). The empowerment of destination residents is deemed crucial for achieving sustainable tourism development (42, 48, 49). Tourism practitioners can leverage digital technologies to analyze tourist preferences via the internet, respond promptly to customer needs, and protect consumer rights (50). Tourists, on the other hand, can not only access travel information but also generate travel content and reviews, thereby enhancing their own capabilities (47).

### 2.5 Smartphone addiction

Smartphone addiction is defined as the excessive use of smartphones despite their negative impacts, taking precedence over other daily activities (51). Earlier research predominantly focused on mobile phone addiction and internet addiction (52, 53). With the shift from mobile phones to smartphones, the term “smartphone addiction” has become more prevalent (54). Smartphone addiction is also referred to as smartphone dependency, overuse, or problematic smartphone use (8).

Characterized by an uncontrollable pattern of excessive smartphone use, smartphone addiction can lead directly or indirectly to a range of issues, such as mental health problems and interpersonal difficulties (55). Due to its convenience and easy internet connectivity, smartphone addiction can be more hazardous than other forms of addiction, making it a significant societal issue (51, 56).

Previous studies on smartphone addiction have primarily focused on children, adolescents, and college students, with relatively few studies on adults, especially older adults (51, 57). The reduction in social roles and weakening of offline relationships among older adults may increase their smartphone usage, potentially leading to smartphone addiction (51).

## 3 Theoretical framework and hypothesis development

### 3.1 Theoretical framework

#### 3.1.1 Self-determination theory

Self-determination theory (SDT) posits that individuals have three basic needs crucial for their survival and development: autonomy, competence, and relatedness (58). When these fundamental needs are satisfied, individuals tend to develop toward health, vitality, and integration; conversely, when these needs are unmet, individuals may experience significant maladaptive issues (58, 59).

Digital literacy plays a pivotal role in facilitating the satisfaction of fundamental needs, thereby promoting enhanced social integration. Online participation transcends temporal and spatial constraints, enabling individuals to autonomously select communication partners and content, thus fulfilling the need for autonomy (60). Individuals with higher digital literacy are more proficient in identifying reliable and secure information, which bolsters their ability and confidence in risk management (61, 62). Furthermore, the internet functions as a powerful tool for fostering close relationships with others, significantly contributing to personal wellbeing (63–65).

#### 3.1.2 Uses and gratifications theory

Uses and gratifications theory (UGT) posits that media choice and usage are purposive and motivated behaviors (66), with users actively seeking to fulfill their personal needs and desires through various uses (67). “Gratification” is the core concept of UGT (66). The internet can provide four types of gratification: content, process, social, and self-presentation (68, 69).

In the initial stages, experienced gratification plays a significant role; however, as gratification shifts toward compensation, experiences of compensation and negative reinforcement become more predominant in the later stages (70). Smartphones have evolved into a one-stop platform capable of satisfying a diverse array of needs (71). Nonetheless, excessive and uncontrolled use of smartphones as a means to compensate for psychological needs can lead to overuse and addiction (72), potentially inducing feelings of guilt (127).

#### 3.1.3 Conceptual model

**Figure 1:**
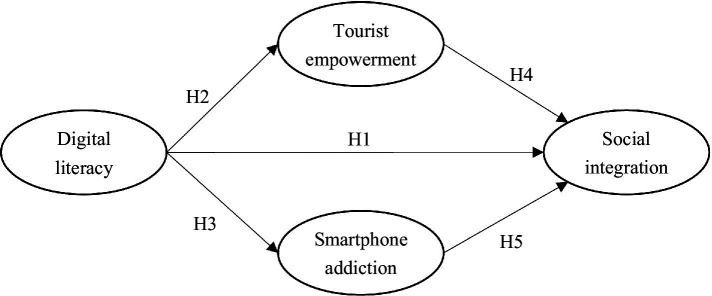
The conceptual model.

According to Self-Determination Theory (SDT), higher digital literacy among retirement migrants enhances their tourist empowerment, thereby more effectively satisfying their needs for autonomy, competence, and relatedness, which subsequently promotes social integration. Conversely, drawing on Uses and Gratifications Theory (UGT), if retirement migrants, after fulfilling these needs, become addicted to smartphones due to a lack of self-control, it may adversely affect their social integration. Consequently, the proposed conceptual model is illustrated in Figure 1.

### 3.2 The impact of digital literacy

#### 3.2.1 The impact of digital literacy on social integration

Digital technology acts as a powerful instrument for fostering the social integration of older adults (73). The impact of digital literacy on social integration can be comprehensively understood through the framework of Self-Determination Theory (SDT). Digital literacy aids in satisfying individuals’ needs for autonomy, competence, and relatedness, thereby exerting a significant influence on the social integration of retirement migrants across three critical dimensions: lifestyle, interpersonal interactions, and activity participation.

Digital literacy profoundly influences lifestyle habits by satisfying the autonomy and competence needs of retirement migrants, enabling them to select preferred lifestyles and effectively manage their lives. Through digital technology, older adults can overcome geographical distances and barriers to physical mobility, thereby enhancing their capacity and confidence to manage their daily activities (74, 75). Moreover, digital literacy enhances individuals’ abilities to acquire, evaluate, and apply health-related information, facilitating easier access to online health resources. This, in turn, contributes to the maintenance and development of healthier lifestyles and travel habits (55, 76, 77).

Digital literacy significantly influences interpersonal interactions by fulfilling the autonomy, competence, and relational needs of retirement migrants. This facilitation of interpersonal interactions is critical as digital literacy enhances older adults ability to communicate with family, friends, and peers, thereby assisting in mitigating loneliness and social isolation in later life (78–80). For instance, retirees can use smartphones to maintain contact with distant friends and develop new social relationships—opportunities that were unavailable to previous generations (74, 75). Additionally, online platforms allow them to autonomously select their communication partners and topics, surpassing spatial and temporal limitations (59, 60). For example, through applications such as WeChat, older adults can effortlessly form interest groups with their peers, exchange various types of information, and actively engage in communal activities (81).

Digital literacy significantly influences activity participation among retirement migrants by fulfilling their autonomy and competence needs. With enhanced digital literacy, retirees can easily access information about community activities, interest groups, health seminars, and more, enabling them to independently select activities and companions that align with their interests (69). Furthermore, digital literacy empowers these individuals to utilize online learning platforms and resources, which continuously enhance their knowledge and skills, thereby increasing their confidence and capacity to engage in various activities (82). A higher level of digital literacy translates into a greater ability for older adults to participate in online activities, leading to improved overall activity engagement. In summary, as older adults become more proficient with technology and recognize its utility in accessing activity-related information, their likelihood of participating in diverse activities correspondingly increases (76, 81).

Based on the aforementioned discussion, we propose the following hypotheses:

*H1*: Digital literacy has a positive impact on the social integration of retirement migrants.

More specifically:

*H1a*: Digital literacy has a positive impact on the lifestyle habits of retirement migrants.

*H1b*: Digital literacy has a positive impact on the interpersonal interaction of retirement migrants.

*H1c*: Digital literacy has a positive impact on the activity participation of retirement migrants.

#### 3.2.2 The impact of digital literacy on tourist empowerment

Digital literacy can mitigate the lifestyle changes associated with aging among older adults, fostering a sense of empowerment (73). Digital literacy enhances tourists’ capabilities to use online platforms and applications for itinerary planning, service booking, and obtaining real-time information (128). This not only increases their sense of control over travel plans but also improves their ability to handle various unexpected situations during their travels.

Social media has shifted the power dynamics between service providers and tourists, adding new dimensions to empowerment (83–85). Digital literacy facilitates tourists in sharing travel experiences and feedback through social media, influencing other tourists’ decision-making processes and amplifying their voice in the travel market (86, 87).

Besides empowering tourists, social media also empowers service providers by enhancing customer targeting (88). Understanding tourists’ needs and expectations more effectively allows travel companies to better cater to their preferences by encouraging them to share and express their thoughts and opinions (89). Therefore, digital literacy plays a crucial role in tourist empowerment.

In summary, we propose the following hypothesis:

*H2*: Digital literacy has a positive impact on tourist empowerment.

#### 3.2.3 The impact of digital literacy on smartphone addiction

The Uses and Gratifications Theory can explain the impact of digital literacy on smartphone addiction. Smartphones can fulfill the social and emotional needs of older adults, especially when they lack interpersonal interactions in real life. Older adults with higher levels of digital literacy are more likely to engage in various online activities, potentially leading to excessive use of digital devices (90, 91). Furthermore, older individuals with a high degree of fear of missing out (FoMO) may overuse smartphones to stay connected, thereby increasing the risk of smartphone addiction (92, 93).

Retirement migrants in unfamiliar environments increasingly rely on digital devices to meet their needs for social interaction, entertainment, information access, and life services (90, 91). Studies have demonstrated that higher digital literacy correlates with longer smartphone usage, and the duration of smartphone use is positively associated with smartphone addiction (51).

In summary, we propose the following hypothesis:

*H3*: Digital literacy has a positive impact on smartphone addiction among retirement migrants.

### 3.3 The impact of tourist empowerment on social integration

Tourist empowerment markedly influences the lifestyle habits of retirement migrants by fostering autonomy and enhancing their ability to manage their lives and make informed decisions (94, 95). This empowerment leads to improved self-reliance and health consciousness, enabling retirement migrants to adopt healthier routines and make better lifestyle choices, ultimately benefiting their overall wellbeing (129).

In the realm of interpersonal interactions, tourist empowerment significantly bolsters the social and emotional support networks of retirement migrants. By improving their ability to communicate and maintain connections with friends, family, or like-minded individuals, these migrants experience enriched social interactions (96, 97).

Finally, tourist empowerment positively influences the participation of retirement migrants in various activities. Increased confidence and reduced anxiety about engaging in new activities lead to higher levels of participation, reflecting positive interpersonal interactions and serving as a direct indicator of social integration (98). Enhanced activity engagement strengthens social bonds and contributes significantly to the migrants’ sense of community and overall social integration.

Therefore, we propose the following hypotheses:

*H4*: Tourist empowerment has a positive impact on the social integration of retirement migrants.

More specifically:

*H4a*: Tourist empowerment has a positive impact on the lifestyle habits of retirement migrants.

*H4b*: Tourist empowerment has a positive impact on the interpersonal interactions of retirement migrants.

*H4c*: Tourist empowerment has a positive impact on the activity participation of retirement migrants.

### 3.4 The impact of smartphone addiction on social integration

Smartphone addiction affects the lifestyle habits of retirement migrants. Smartphone addiction adversely affects both physical and mental health (99). For example, People frequently use smartphones while eating, during breaks, and even place them next to their pillows while sleeping, which can lead to problematic lifestyle habits (7, 100).

Smartphone addiction affects the interpersonal interactions activity participation of retirement migrants. The internet facilitates individuals’ absorption in the virtual world at the expense of engaging with the physical world (96). The lack of meaningful virtual interactions can hinder the establishment of genuine, deep relationships, thus impeding social integration (52). For instance, excessive reliance on smartphone interactions instead of engaging directly with family and friends detracts from more direct and authentic physical and social experiences, leading to isolation from the real world (101, 102). Furthermore, smartphone addiction can result in reduced participation in offline activities and the degradation of social skills (103).

In summary, we propose the following hypotheses:

*H5*: Smartphone addiction has a negative impact on the social integration of retirement migrants.

More specifically:

*H5a*: Smartphone addiction has a negative impact on the lifestyle habits of retirement migrants.

*H5b*: Smartphone addiction has a negative impact on the interpersonal interactions of retirement migrants.

*H5c*: Smartphone addiction has a negative impact on the activity participation of retirement migrants.

## 4 Methodology

### 4.1 Study sites

This study focused on two distinct regions in China: Hangzhou in Zhejiang Province, and Hainan Province (specifically Chengmai County and Ledong Li Autonomous County). The selection of these locations ensures sample representativeness and diversity, capturing both urban and resort-like environments that reflect different retirement migration settings. Hangzhou, famously described as part of “heaven on earth” alongside Suzhou, is not only an internationally renowned tourist destination but also a popular choice for retirement migrants. Hainan, with its significant retiree population, has become a focal point for retirement migration research, attracting considerable academic attention (12).

### 4.2 Participants

Participants were recruited from retirement communities and senior living facilities in both locations, focusing on areas with high concentrations of retirement migrants. The study employed a convenience sampling method to recruit individuals aged 50 and above who were in good health. The age threshold of 50 was chosen based on two key considerations: age provides a straightforward and objective criterion, making it easier to define compared to more subjective standards; and individuals aged 50 and above are more likely to exhibit common signs of aging, such as gray hair, physical weakness, wrinkles, and declining functional abilities, making them more representative of the retirement migrant population (104).

While convenience sampling was used due to resource constraints and accessibility issues, steps were taken to minimize potential bias. These included recruiting from various retirement communities and facilities to ensure diversity in socioeconomic status and lifestyle preferences, and employing quota sampling to maintain a balanced representation of age groups and genders. These measures aimed to enhance the sample’s representativeness despite the limitations of the convenience sampling method.

### 4.3 Measurement instrument

The measurement scales utilized in this study were primarily adapted from established scales in previous research. As the original scales were developed in English, a rigorous translation and back-translation procedure was implemented to ensure cultural and linguistic equivalence. Two bilingual experts independently translated the scales into Chinese, with any discrepancies resolved through collaborative discussion. Subsequently, a third expert back translated the Chinese version to English to verify accuracy and ensure conceptual equivalence across languages. Two bilingual experts independently translated the scales, with discrepancies resolved through collaborative discussion. A third expert then back translated the Chinese version to English to verify accuracy and ensure conceptual equivalence across languages.

The final questionnaire comprises two main sections. The first section gathers demographic information, including gender, age, education level, and income. The second section consists of a 21-item measurement scale assessing four key constructs: digital literacy, tourist empowerment, smartphone addiction, and social integration. These constructs are measured using a 5-point Likert scale, with responses ranging from “1” (strongly disagree) to “5” (strongly agree) for digital literacy, tourist empowerment, and social integration. Smartphone addiction is assessed on a frequency scale from “1” (never) to “5” (always). All scales have been linguistically adapted to suit the research context.

*Independent variable*. Digital literacy: recognizing the limitations of previous instruments that often measured only one or two components of the DigComp framework (105), this study employs a four-item scale specifically designed to assess digital literacy among older adults (106, 107). A sample item is: “I can effectively find the information I want on the internet through my smartphone.”

*Mediating Variables*. (a) Tourist empowerment. Measured using three items adapted from the Tourist Consumer Empowerment Scale (108). An example item is: “I can use various means to obtain a better experience in retirement tourism.” (b) Smartphone addiction. Assessed with four items adapted from the Mobile Phone Dependency Scale (52, 109). A representative item is: “People around me have complained about my smartphone use.”

*Dependent variable*. Social integration: drawing on established Social Integration Scales (2, 18), this construct is measured across three dimensions - “lifestyle habits, ““interpersonal interactions, “and “activity participation” - using a total of 10 items.

### 4.4 Data collection

Data collection for this study was conducted from January 15, 2024, to January 30, 2024. To ensure consistency and accuracy throughout the process, two research assistants were assigned to manage data collection at each survey location. Prior to initiating the survey, all participants were provided with a detailed explanation of the study’s purpose, potential risks and benefits, and their rights as research subjects. The questionnaires were administered face-to-face, allowing participants to ask questions and receive clarification from the research assistants throughout the completion process. To protect participants’ privacy, all responses were collected anonymously, with no personally identifiable information gathered. A reminder mechanism was also employed during the data collection period to improve response rates.

A total of 500 questionnaires were distributed, of which 425 were returned. After data cleaning—removing responses with low completion rates, internal contradictions, or missing key information—369 valid responses were included in the final analysis, resulting in an effective response rate of 73.8%. All data were securely stored, with access restricted to the research team to maintain confidentiality and data integrity.

### 4.5 Data analysis

This study primarily employs two statistical analysis software packages: IBM SPSS Statistics 23.0 and AMOS 23.0, to analyze the questionnaire survey data. Firstly, descriptive analysis of the questionnaire data is conducted using SPSS, and the common method bias of the data is tested. Secondly, structural equation modeling (SEM) is performed using AMOS software to test the research hypotheses through path analysis. Lastly, the bootstrap method is utilized to examine the mediating effects, specifically verifying the mediating roles of tourist empowerment and smartphone addiction.

## 5 Results

### 5.1 Descriptive data analysis

A total of 369 respondents participated in this survey. The demographic characteristics of the respondents are shown in Table 1. There were 224 females and 144 males, with more female respondents than male respondents. Regarding the age distribution of the respondents, the survey mainly targeted older adults population aged 50 and above, with a significant concentration in two age groups: 61–70 years old and 71–80 years old, accounting for 32.5 and 31.4%, respectively. In terms of educational attainment, the majority of respondents held a bachelor’s degree, with 169 individuals, accounting for 45.8%, followed by those with a high school or vocational diploma, totaling 126 individuals, accounting for 34.1%. The monthly income of the respondents was primarily concentrated below 7,000 CNY, with 149 individuals earning <5,000 Yuan, accounting for 40.4%, and 128 individuals earning between 5,000 and 7,000 Yuan, accounting for 34.7%.

**Table 1:** Profiles of respondents (*N* = 369).

Variables	Frequency	%
Gender		
Male	144	39.0
Female	224	60.7
Missing	1	0.3
Age		
<60	38	10.3
61–70	120	32.5
71–80	116	31.4
81–90	71	19.2
>90	17	4.6
Missing	7	1.9
Education		
Junior high school and below	66	17.9
Senior high school or secondary school	126	34.1
Bachelor’s degree	169	45.8
Master’s degree or above	7	1.9
Missing	1	0.3
Income (CNY)		
Under 5,000	149	40.4
5001–7,000	128	34.7
7,001–1,0000	66	17.9
1,001–15,000	19	5.1
15,001–20,000	2	0.5
>20,000	2	0.5
Missing	3	0.8

Data normality was assessed by examining skewness and kurtosis values, as indicated in Table 2. The skewness values ranged from −1.304 to 0.635, while the kurtosis values ranged from −1.258 to 2.459. Following the thresholds suggested by Kline (110) of skewness <3 and kurtosis <8, it was determined that the data exhibited normal distribution.

**Table 2:** Normal distribution test.

Variable	Item	Mean		Skewness		Kurtosis	
		Statistic	Std. error	Statistic	Std. error	Statistic	Std. error
Digital literacy	DL1	4.13	0.050	−1.076	0.127	0.673	0.253
	DL2	4.16	0.052	−1.285	0.127	1.291	0.253
	DL3	3.91	0.056	−0.959	0.127	0.352	0.253
	DL4	4.04	0.059	−1.227	0.127	0.803	0.253
Tourist empowerment	TE1	3.73	0.054	−0.592	0.127	−0.178	0.253
	TE2	3.45	0.057	−0.351	0.127	−0.412	0.253
	TE3	3.62	0.058	−0.601	0.127	−0.272	0.253
Smartphone addiction	SA1	2.40	0.068	0.635	0.127	−0.698	0.253
	SA2	2.61	0.070	0.418	0.127	−1.027	0.253
	SA3	3.07	0.066	−0.038	0.127	−0.989	0.253
	SA4	2.84	0.073	0.163	0.127	−1.258	0.253
Lifestyle habits	SI1	4.28	0.038	−1.021	0.127	1.468	0.253
	SI2	3.60	0.058	−0.553	0.127	−0.441	0.253
	SI3	4.18	0.040	−0.945	0.127	1.223	0.253
Social interaction	SI4	4.05	0.049	−1.016	0.127	0.734	0.253
	SI5	4.17	0.044	−0.996	0.127	0.754	0.253
	SI6	3.95	0.056	−0.855	0.127	−0.081	0.253
	SI7	4.41	0.036	−1.304	0.127	2.459	0.253
Activity participation	SI8	4.08	0.053	−1.103	0.127	0.684	0.253
	SI9	3.58	0.064	−0.534	0.127	−0.751	0.253
	SI10	3.66	0.063	−0.769	0.127	−0.327	0.253

### 5.2 Common method bias

Since all items in this study were completed by retirement migrants, there is a possibility of a common method bias issue. To examine the presence of common method bias in this study, the Harman single-factor test method was employed to assess the data homogeneity. The first common factor accounts for 32.36% of the total load when not rotated, which falls below the standard threshold of 40% (130). This suggests that there is no significant common method bias in this study, allowing for further data analysis to proceed.

### 5.3 Measurement model

A confirmatory factor analysis (CFA) employing MLM estimation was conducted using AMOS 23.0 to evaluate the construct validity and reliability of the proposed model. Table 3 outlines the variables and their corresponding items. The Cronbach’s alpha (*α*) coefficients for the six latent variables all surpass the recommended threshold of 0.7suggested by Churchill (111), except for Lifestyle habits. Additionally, the composite reliability (CR) scores for each latent variable exceed the suggested level of 0.70, affirming the internal consistency reliability of the measurement items. Convergent validity of the measures is supported by average variance extracted (AVE) values surpassing the recommended threshold of 0.50 for each construct, except for Lifestyle habits. Lastly, the measurement model exhibits a favorable fit to the data: *χ*^2^/df = 1.861, GFI = 0.929, CFI = 0.970, NFI = 0.937, TLI = 0.961, RMSEA = 0.048. These metrics collectively indicate that the model is well-suited to the observed data (112).

**Table 3:** Results of confirmatory factor analysis (*N* = 369).

Variable	Item	Estimate	*P*	Cronbach’s alpha	CR	AVE
Digital literacy	DL1	0.846	***			
	DL2	0.856	***	0.912	0.908	0.712
	DL3	0.841	***			
	DL4	0.833	***			
Smartphone addiction	SA1	0.806	***			
	SA2	0.919	***	0.894	0.880	0.649
	SA3	0.757	***			
	SA4	0.727	***			
Tourist empowerment	TE1	0.771	***	0.856	0.857	0.666
	TE2	0.867	***			
	TE3	0.808	***			
Social integration						
Lifestyle habits	SI1	0.566	***	0.698	0.676	0.415
	SI2	0.766	***			
	SI3	0.580	***			
Social interaction	SI4	0.852	***			
	SI5	0.843	***			
	SI6	0.837	***	0.867	0.879	0.648
	SI7	0.675	***			
Activity participation	SI8	0.676	***			
	SI9	0.896	***	0.846	0.854	0.664
	SI10	0.856	***			

*** < 0.001.

Discriminant validity was evaluated by contrasting the intercorrelations of the variables with the square root of the average variance, as proposed by Petrick (113). Table 4 displays the findings. The shared variance between variable pairs was found to be lower than the corresponding Average Variance Extracted (AVE) values, except for Lifestyle habits, consistent with the criteria outlined by Fornell and Larcker (114), indicating satisfactory discriminant validity.

**Table 4:** Discriminate validity analysis from CFA.

	Digital literacy	Smartphone addiction	Tourist empowerment	Lifestyle habits	Social interaction	Activity participation
Digital literacy	**0.844** ^**a**^					
Smartphone addiction	0.347^b^	**0.806**				
Tourist empowerment	0.242	0.391	**0.816**			
Lifestyle habits	0.284	0.132	0.575	**0.644**		
Social interaction	0.257	0.148	0.377	0.651	**0.805**	
Activity participation	0.392	0.277	0.611	0.610	0.530	**0.815**

^aBold diagonal values: square root of the variance that is common between the factors and their respective measures. bValues below diagonal are the correlations between factors. To establish discriminant validity, the diagonal values should exceed any other corresponding entries in their respective rows or columns.^

### 5.4 Structural model

AMOS 23.0 was utilized to conduct Structural Equation Modeling (SEM) to estimate the main effects model, with its parameters utilized to test all the hypotheses. Additionally, the main effects hypotheses were assessed through SEM. The results of SEM demonstrated a satisfactory overall fit: *χ*^2^/df = 1.917, TLI = 0.959; CFI = 0.968; NFI = 0.935; GFI = 0.927; IFI = 0.968; RMSEA = 0.050.

As demonstrated in Table 5, the SEM results also indicated that elders’ digital literacy has a significant positive effect on their social integration, Lifestyle habits (*β* = 0.198, *p* < 0.05), Social interaction (*β* = 0.194, *p* < 0.05), and activity participation (*β* = 0.269, *p* < 0.05), thus, H1a, H1b and H1c were supported. At the same time, the SEM results indicated that elders’ digital literacy has a significant positive effect on Smartphone addiction (*β* = 0.394, *p* < 0.05) and Tourist empowerment (*β* = 0.245, *p* < 0.05), H2 and H3 were supported. Additionally, Tourist empowerment has a significant positive effect on their Lifestyle habits (*β* = 0.533, *p* < 0.05), Social interaction (*β* = 0.364, *p* < 0.05) and activity participation (*β* = 0.558, *p* < 0.05), thus, H4a, H4b and H4c were accepted. Smartphone addiction has a significant negative effect on Lifestyle habits (*β* = −0.183, *p* < 0.05), and has no significant effect on social interaction (*β* = −0.081, *p* > 0.05), and activity participation (*β* = −0.034, *p* > 0.05), thus, H5a was accepted, but H5b, H5c were rejected.

**Table 5:** Hypothesis testing.

Path	Estimate	SE	T	P	Results
Digital literacy → Smartphone addiction	0.294	0.067	4.940	***	Yes
Digital literacy → Tourist empowerment	0.245	0.053	4.059	***	Yes
Digital literacy → Lifestyle habits	0.198	0.034	3.080	0.002	Yes
Digital literacy → Social interaction	0.194	0.051	3.313	***	Yes
Digital literacy → activity participation	0.269	0.041	4.902	***	Yes
Smartphone addiction → Lifestyle habits	−0.183	0.028	−3.006	0.003	Yes
Smartphone addiction → Social interaction	−0.081	0.041	−1.515	0.130	No
Smartphone addiction → Activity participation	−0.034	0.031	−0.723	0.470	No
Tourist empowerment → Lifestyle habits	0.533	0.047	6.930	***	Yes
Tourist empowerment → Social interaction	0.364	0.063	5.707	***	Yes
Tourist empowerment → Activity participation	0.558	0.058	8.319	***	Yes

*** < 0.001.

### 5.5 Mediation effects

We further investigated the mediating effects of Tourist Empowerment (TE) and Smartphone addiction (SA) using the methodology advocated by Zhao et al. (115). Results are presented in Tables 6. In the bootstrapping analysis, a path is deemed significant and corroborated if the bootstrap confidence interval excludes zero. First of all, tourist empowerment (*β* = 0.065, *p* < 0.05, CI = [0.031, 0.114]) and smartphone addiction (*β* = −0.025, *p* < 0.05, CI = [−0.058, −0.004]) play a significant role in the relationship between digital literacy and Lifestyle habits, given the direct effect is significant, thus, tourist empowerment and Smartphone addiction are partial mediator. Secondly, tourist empowerment (*β* = 0.073, *p* < 0.05, CI = [0.031, 0.128]) play a significant role in the relationship between digital literacy and social interaction given the direct effect is significant, thus, tourist empowerment plays a partial mediator role in the relationship between digital literacy and social interaction. However, the mediating effect of Smartphone addiction is not significant. Finally, tourist empowerment (*β* = 0.102, *p* < 0.05, CI = [0.045, 0.171]) play a significant role in the relationship between digital literacy and activity participation, given the direct effect is significant, thus, tourist empowerment plays a partial mediator role in the relationship between digital literacy and activity participation. However, the mediating effect of Smartphone addiction is not significant.

**Table 6:** The results of mediation effect test.

Path		Estimate	*P*	95% Confidence Interval	
				BC	
				Lower	Upper
Digital literacy (DL)-Lifestyle habits (LH)					
Indirect effect	DL- SA -LH	−0.025	0.029	−0.058	−0.004
Indirect effect	DL-TE-LH	0.065	0.000	0.031	0.114
Direct effect	DL-LH	0.103	0.013	0.021	0.189
Total effect		0.142	0.001	0.072	0.222
DL-Social interaction (SI)					
Indirect effect	DL-SA-SI	−0.008	0.692	−0.048	0.038
Indirect effect	DL-TE-SI	0.073	0.001	0.031	0.128
Direct effect	DL-SI	0.159	0.024	0.031	0.284
Total effect		0.223	0.001	0.111	0.344
DL-activity participation (AP)					
Indirect effect	DL- SA -AP	−0.007	0.638	−0.042	0.022
Indirect effect	DL-TE-AP	0.102	0.001	0.045	0.171
Direct effect	DL-AP	0.206	0.001	0.104	0.325
Total effect		0.301	0.001	0.196	0.418

TE, tourist empowerment; SA, smartphone addiction; BC, bias-corrected and accelerated; bootstrap = 2000.

## 6 Discussion

In the context of the intertwining trends of aging and digitalization, the social integration of retirement migrants faces new opportunities and challenges, necessitating increased research attention. This study, grounded in Self-Determination Theory and Uses and Gratifications Theory, has constructed a parallel mediation model to uncover the relationship between digital literacy and social integration among retirement migrants. Several notable findings from this research warrant further discussion.

1. *Digital literacy positively influences the social integration of retirement migrants.*

This finding is consistent with previous research. According to Self-Determination Theory, digital literacy meets the needs for autonomy, competence, and relatedness among retirement migrants, thereby promoting their social integration (58). Learning to use new technologies can assist older adults in integrating into today’s digital society (116).

Digital literacy enhances the lifestyle habits of retirement migrants, aligning with previous research findings. The higher the digital literacy of retirement migrants, the more effectively they can gather useful and comprehensive health information, leading to the formation of autonomous and controlled healthy lifestyle habits (55, 76, 77).

Additionally, digital literacy fosters interpersonal interactions among retirement migrants, consistent with existing studies. Higher digital literacy enables retirement migrants to access online informational and emotional support more effectively. In environments rich in informational and emotional support, individuals are more inclined to engage in information exchange, emotional communication, mutual assistance, and the development of friendships (117–119). Furthermore, digital literacy facilitates richer interpersonal interactions for older adults during travel (75, 120).

Moreover, digital literacy promotes activity participation among retirement migrants, as corroborated by previous research. The higher the digital literacy of retirement migrants, the more access they have to information about activities, thereby enhancing their capability to engage in them (76, 81).

2. *Tourist empowerment plays a mediating role in the positive influence of digital literacy on the social integration of retirement migrants.*

This finding is consistent with both previous research and our expectations. According to Self-Determination Theory, digital literacy enhances the capabilities of retirement migrants, fulfilling their need for “competence” and thereby promoting social integration. The use of information and communication technology by older adults helps increase their sense of control and independence in daily life, strengthen social networks, and participate in leisure and other meaningful activities (94, 121).

Digital literacy enhances the lifestyle habits of retirement migrants though tourist empowerment, consistent with existing research. Higher levels of digital literacy enable retirement migrants to utilize various digital technologies to access information and resources, facilitating more informed lifestyle choices (95, 131). For example, using smartphones and social media empowers consumers by providing easier access to vast amounts of information, thereby enhancing their decision-making capabilities (45).

Digital literacy also promotes interpersonal interactions among retirement migrants through tourist empowerment, aligning with previous studies. Digital literacy helps retirement migrants overcome temporal and spatial constraints, allowing for interpersonal interactions anytime and anywhere (37, 38). Additionally, digital literacy can enrich the content and forms of interpersonal interactions. Smartphones enable retirement migrants to generate and share content at any time, exchanging ideas and opinions with online peers (87).

Furthermore, digital literacy enhances the participation in activities of retirement migrants by empowering tourists, in accordance with existing research. Digital literacy improves their ability to effectively use digital tools such as travel review websites, navigation applications, and social media. These tools have become indispensable for planning, experiencing, and sharing travel experiences (128). The use of digital tools in tourism activities can enhance both satisfaction and engagement (132).

3. *The negative mediating effect of smartphone addiction is partially confirmed.*

Specifically, it has a significant negative mediating impact on the relationship between digital literacy and social integration in the “lifestyle habits” dimension, but does not have a significant negative mediating effect on the “interpersonal interaction” and “activity participation” dimensions of social integration. The mediating effect of smartphone addiction was partially validated, which is not entirely consistent with the anticipated research.

The positive impact of digital literacy on smartphone addiction was confirmed, aligning with previous studies. This phenomenon can be explained by the Uses and Gratifications Theory (UGT), which suggests that after using smartphones to meet their needs, individuals may become addicted if their usage is not controlled. Studies have shown a positive correlation between digital literacy and excessive smartphone dependency (51). Specifically, higher levels of digital literacy are associated with greater smartphone dependency, resulting in more frequent smartphone use (122).

The negative impact of smartphone addiction on the lifestyle habits dimension of social integration among retirement migrants is consistent with previous research. Studies have shown that excessive reliance on smartphones can lead to adverse consequences such as distraction, sleep disorders, and decreased productivity (103, 123). However, the negative impact of smartphone dependency on the interpersonal interactions and activity participation dimensions of social integration among retirement migrants is inconsistent with previous research. Previous studies have suggested that smartphone addiction causes individuals to become engrossed in the online virtual world, thus neglecting more direct and tangible physical and social interactions in the offline world (96, 102). This inconsistency may be attributed to the different types of smartphone usage.

Smartphones have different usage types, which can be categorized into information seeking, entertainment seeking, communication seeking, and life services (103, 124). Entertainment seeking and life services usage types are positively correlated with smartphone addiction, whereas information seeking and communication seeking are negatively correlated with smartphone addiction (51). This distinction is further supported by studies on internet usage (IU). Research has shown that entertainment-oriented IU positively affects internet addiction; the more an individual engages in entertainment-oriented IU, the higher their degree of internet addiction. Conversely, educational-oriented IU negatively affects internet addiction; the more educational-oriented IU, the lower the degree of internet addiction (125). The way retirement migrants use smartphones differs significantly from that of teenagers. Retirees primarily use smartphones for information gathering and interpersonal communication, rather than for games and entertainment. Consequently, smartphone addiction does not diminish offline social activities.

## 7 Conclusion

### 7.1 Main findings

This research seeks to explore the dual effects and mediating pathways through which digital literacy influences social integration among retirement migrants. Based on the results and discussion, the research conclusion can be summarized as follows: digital literacy demonstrates a double-edged sword effect on the social integration of retirement migrants. Specifically, digital literacy facilitates social integration through tourist empowerment while impeding it through smartphone addiction. These findings underscore the paradoxical “empowerment-alienation” dynamic inherent in technology-enabled aging processes.

### 7.2 Implications

#### 7.2.1 Theoretical implications

Firstly, this study introduces digital literacy as an antecedent variable, expanding the factors influencing social integration from an individual level. By revealing the impact mechanism of digital literacy on social integration, this study enriches and extends the research in the field of social integration. Previous studies have predominantly examined the social integration of retirement migrants from the perspectives of the tourism destination environment and interactions with local residents, with limited exploration from the individual perspective of the retirees themselves.

Secondly, this study further elucidates the formation mechanism of social integration among retirement migrants. By introducing tourist empowerment and smartphone addiction as parallel mediating variables, this research explores the impact mechanism of digital literacy on social integration from different perspectives. On one hand, by integrating the characteristics of retirement migrants, this study further confirms that tourist empowerment serves as a crucial pathway linking digital literacy and social integration. On the other hand, the research reveals that smartphone addiction exerts a certain inhibitory effect on the social integration of retirement migrants, providing valuable insights for subsequent studies on social integration at a theoretical level.

Thirdly, this study achieves an effective integration of the Self-Determination Theory and the Uses and Gratifications Theory. By jointly introducing these two theories into the research on the relationship between digital literacy and social integration, this study not only expands the application scope of the aforementioned theories but also provides new theoretical foundations for exploring social integration among retirement migrants. The empirical results of this study indicate that at the individual level, digital literacy can empower retirement migrants. Social integration requires not only empowerment through digital literacy but also the prevention of smartphone addiction.

#### 7.2.2 Managerial implications

This study examines the impact mechanism of social integration from the perspective of digital literacy. It aims to promote the social integration of retirement migrants through empowering tourists and preventing smartphone addiction. This novel mechanism provides beneficial practical insights for government departments, tourism enterprises, and retirement migrants themselves.

Firstly, government departments and tourism enterprises should collaborate with various stakeholders to enhance the digital literacy of older adults. Digital literacy can promote social integration. Therefore, on one hand, it is essential to construct an age-friendly digital society by renovating or developing digital platforms or tools tailored for older adults, providing convenience for their learning and usage. On the other hand, older adults should receive training in digital knowledge and skills, with a particular focus on using smartphones and social media, to enhance their perception of the utility and convenience of digital technology. Encouraging intergenerational support through digital knowledge transfer from younger generations to older adults should also be promoted.

Secondly, government departments and tourism enterprises should leverage digital technology to empower tourists, harnessing the positive reinforcement of tourist empowerment on social integration. For instance, utilizing digital platforms such as short videos and tourism websites to provide online information services for retirement migrants. Encouraging retirement migrants to utilize digital applications like health management and social media to enhance life management, engage in social interactions, and participate in recreational activities would be beneficial.

Thirdly, on an individual level, retirement migrants should guard against excessive use of digital tools and cultivate healthy lifestyle habits. Smartphone addiction has a certain inhibitory effect on social integration. Therefore, while enhancing digital literacy, retirement migrants should pay attention to time management and enhance self-control to prevent addiction to smartphones. Additionally, they should be mindful of the types of smartphone usage, prioritizing information retrieval, accessing life services, and engaging in interpersonal communication, while minimizing usage for gaming and entertainment purposes.

### 7.3 Limitations and future directions

The investigation into the relationship between digital literacy and social integration among retirement migrants is a relatively novel research domain, and this study presents several limitations that highlight areas for future inquiry.

Firstly, the sample exclusively comprised Chinese retirement migrants, which may restrict the generalizability of the findings to other cultural contexts. Future research should consider extending the sample to include individuals from diverse cultural backgrounds to assess the cross-cultural applicability of the results.

Secondly, the utilization of cross-sectional data in this study constrains the capacity to infer causal relationships between variables. Implementing longitudinal data or experimental designs, in future research would furnish more compelling evidence to elucidate the causal mechanisms between variables and offer a deeper understanding of the evolution of digital literacy and social integration over time.

Thirdly, other mediating factors may influence the relationship between digital literacy and social integration. Future research should explore alternative transmission mechanisms from various theoretical perspectives. Additionally, examining the role of moderating variables could provide a more nuanced understanding of this relationship.

## Data Availability

The raw data supporting the conclusions of this article will be made available by the authors, without undue reservation.
